# Influencing Factors of Sulfuric Acid Resistance of Ca-Rich Alkali-Activated Materials

**DOI:** 10.3390/ma16062473

**Published:** 2023-03-20

**Authors:** Zhuguo Li, Ko Ikeda

**Affiliations:** Graduate School of Science and Technology for Innovation, Yamaguchi University, Tokiwadai, Ube 755-8611, Yamaguchi, Japan

**Keywords:** alkali-activated material, acid resistance, chemical admixture, inactive precursor, waste incineration ash

## Abstract

In this paper, we distinguished the degradation of alkali-activated material (AAM) exposed to sulfuric acid as physical (scaling, spalling, cracking, breaking, etc.) and chemical degradation (neutralization), because the mechanisms of these two types of degradation are different. Then, the effects of curing method, raw materials, and their mixing proportions on the two kinds of degradation of AAMs containing GGBFS were investigated in detail, including liquid-filler ratio, component of alkali activator, chemical admixture, inactive filler alternative to fly ash (FA), addition of municipal waste incineration bottom ash (BA), etc. The experimental results show that (a) small liquid-filler ratio, heat-curing, and the use of blended alkali activator solution of sodium silicate and NaOH can reduce both physical and chemical degradation of AAMs; (b) large GGBFS content or AE agent addition decreases the physical degradation, but increases the chemical degradation; (c) using crushed stone powder to replace FA and adding BA or a retarder would increase the physical and chemical degradation; but (d) the use of drying shrinkage reducer composed of polyether derivatives does not affect acid resistance. We also discussed the applicability and limitation of XRD and SEM-EDS in analyzing the chemical compositions of Ca-rich AAMs exposed to sulfuric acid, and found that (e) XRD analysis can identify the gypsum formation, and the gypsum peak intensity is related to the physical degradation of the Ca-rich AAMs; (f) by SEM-EDS analysis, the decalcification and dealkalization of C-A-S-H gels can be judged from the decrease in the average Ca/Si atomic ratio and the average Na atomic percentage in the acid corrosion area, but dealumination can be only determined from the dissimilarity of Al and Si elemental maps; and (g) if the CaO/SO_3_ molar ratio ranges from 0.8 to 1.0, gypsum formation can be estimated.

## 1. Introduction

Concretes in hot spring areas, acid river basins, and sewage systems, in contact with sulfate groundwater, seawater, and soil, and in plants where acids are used, are susceptible to acid and sulfate attack. Currently used for concretes, Portland cement (PC) and blended cement has low acid resistance. Sulfate attack on PC concrete is often said to arise from each of two major sulfate reactions: (1) the sulfate ions react with C_3_A and its hydration products to form ettringite, with an increase in volume that results in expansion and subsequent cracking of the concrete; (2) the sulfate ions react with calcium hydroxide (CH) to form gypsum, which is less soluble in water and becomes deposited in the voids, causing internal stresses and leading to disruption and strength loss of the matrix [1,2]. 

There are two generally used ways to improve concrete’s resistance to acids: (a) choosing the right concrete composition to make it as impermeable as possible, e.g., using sulfate-resisting cement, which has a lower content of C_3_A; and (b) isolating it from the aggressive environment by using a suitable coating, such as epoxy coating or nanocoating [3,4]. Obviously, these methods require high costs, and the coating methods have degradation challenges.

Sulfuric acid is found in sewer pipes due to the reaction between hydrogen sulfide and oxygen in the presence of aerobic sulfur-oxidizing bacteria [5]. In Japan, the standard service life of PC concrete sewage pipes is 50 years. The costs for repairing and renewing sewerage facilities were estimated to be approximately JPY 0.8 trillion/year in 2008 but will increase to approximately JPY 1.0 trillion/year in 2023 and about JPY 1.2–1.3 trillion/year in 2028 [6]. Therefore, there is a need to develop acid-resistant concrete for sewerage systems and the concrete structures located in acidic environments.

In recent decades, the cement and concrete industry has been under increased pressure to reduce CO_2_ emissions and the consumption of natural resources. This has led to an increased attention to alkali-activated binders or geopolymers, which use aluminosilicate source and alkali activator rather than limestone-derived clinker, and thus has a low CO_2_ emission intensity [7]. Fly ash (FA) and ground granulated blast furnace slag (GGBFS) are usually used as aluminosilicate sources, because they are wastes from coal-fired power generation and steel production, respectively. Alkali-activated FA/GGBFS blends can be cured at ambient temperature and are less susceptible to fast settings [8,9]. Therefore, blended FA/GGBFS systems (AAFS) have generated significant interest in recent years. Alkali-activated slag (AAS), AAFS, and other AAMs using GGBFS and other fillers [10] can be considered as Ca-rich alkali-activated materials.

Most of the studies [11,12] on acid attack of AAMs have found that AAMs have better acid resistance than PC. Significant acid resistance is usually attributed to decalcified and modified aluminosilicate gel formation after an acid attack [13]. Dealumination was confirmed as the dominating microstructural change of synthetic N-A-S-H gels under an H_2_SO_4_ attack [14]. The exchange between alkali cations (Na^+^, K^+^) and hydronium ions (H_3_O^+^), called dealkalization, is thought to lead to the destabilization of aluminosilicate gels [13].

The C-A-S-H gels in alkali-activated binders with rich Ca suffer both decalcification and dealumination, leaving behind silicon-rich gels, which help resist further acid attacks [15]. Decalcification is the main mechanism of deterioration [16]. The deteriorating properties of AAMs exposed to strong acids are caused by an initial exchange between alkali and alkaline earth cations (Na^+^, K^+^, Ca^2+^, and Mg^2+^) and hydronium ions (H_3_O^+^). The initial exchange between alkali and alkaline earth cations (Na^+^, K^+^, Ca^2+^, and Mg^2+^) and hydronium ions (H_3_O^+^) leads to the decalcification of the binder and the formation of soluble salts, namely calcium acetate, when exposed to acetic acid, or gypsum, when exposed to sulfuric acid [13]. Mass gain caused by gypsum formation may only occur when a small amount has formed. The formation of gypsum causes expansion and internal cracks. Once sufficient expansion stress is caused, the material is likely to suffer mass loss instead of gain and surface corrosion [14]. The surface deterioration is predominantly caused by the combined decalcification and dealumination of calcium sodium aluminosilicate hydrate (C-(N)-A-S-H) gels, and increasing the slag content of AAFS decreases porosity but makes the reaction products more susceptible to sulfuric acid attack [17]. Gypsum can also act as a barrier to further acid attacks. Therefore, the acid resistance test results need careful interpretation and cannot be correlated with the performance of other acid types.

Lloyd et al. [18] investigated the effect of slag addition on AAF pastes exposed to H_2_SO_4_. After 28 days of immersion, the corroded depth of the alkali-activated FA (AAF) pastes was approximately 6 mm, which was reduced below 3 mm with 50% substitution of fly ash for slag. The reduced corrosion depth was explained by a reduction in permeability and pore size due to the addition of slag. Lee and Lee [19] studied the resistance of different fly ash blends and slag to an H_2_SO_4_ (10%) attack. They reported that after 56 days of immersion, AAFS with 0, 10, and 30% slag displayed negligible mass changes. However, when the binder contained 50% slag, a mass gain was observed. Therefore, they concluded that there are two causes of deterioration in AAFS due to the H_2_SO_4_ attack. The first one is corrosion utilizing sulfate penetration, which is associated with permeable voids and the water absorption rate. The second is the corrosion of the reaction products (calcium aluminosilicate hydrate gels), which depend on the fly ash/slag ratio.

Fernandez-Jimenez et al. [20] investigated the effect of different activator solutions on the resistance of AAF to HCl (pH = 1.0) for 90 days. The different activators displayed very similar visual appearance and strength loss results. However, the samples activated with only sodium hydroxide (8 mol/L) displayed a mass loss of 2.5%, compared with a mass loss of 4.2% for the samples activated with a blend of sodium hydroxide (12.5 mol/L, 85%) and sodium silicate (SiO_2_/Na_2_O = 0.16, 15%) solutions. Bakharev [21] found alkali-activated (class F) FA, which was prepared with NaOH and cured at elevated temperature, presented better against acid attack than other activators (sodium silicate, a blend of NaOH and KOH) when exposed to 5% solutions of acetic and sulfuric acids. AAF mortar specimens activated by a blended activator of NaOH and sodium silicate solutions with higher alkali content (Na_2_O) were observed to lose weight and have a faster rate of dealkalization, but show very little loss in strength, in 10% sulfuric acid solution [22]. The effect of the activator content was already studied on AAF [17]. It was also found that increasing the alkali activator dosage of AAF has little impact on sulfuric acid resistance. However, no studies reported the effect of alkali activator dosage on AAFS’s acid resistance, and the effect of the curing regime on acid resistance was also rarely considered [23]. There is no study to be found on how chemical admixture affects the acid resistance of AAMs.

Takagaito & Li, et al. discussed the effects of the blending ratio of GGBFS, fineness of active fillers (FA and GGBFS), components of the alkali activator solution (AS), curing temperature, etc., on the acid resistance of AAFS concrete using river sand and limestone crushed stone through the measurements of mass reduction and corrosion depth [24]. However, the measuring results were inevitably influenced by the aggregates. These influences include the aggregate corrosion and the constraint of aggregate on the variation of the AAFS matrix’s dimension.

Degradation of AAM after acid attack includes spalling, breakage, cracking, strength loss, alkaline reduction, etc. These degradation behaviors occur by different mechanisms and need to be considered separately when exploring their respective influencing factors. Though the excellent acid resistance of AAFS has been well confirmed, detailed investigation of the factors influencing the degradation of AAFS in acidic environments has been lacking. In this study, we investigated the factors influencing acid resistance of AAFS subjected to sulfuric acid attack, for physical and chemical degradation, including AS components, liquid-filler ratio, curing method, and chemical admixtures (retarder, AE agent, and drying shrinkage reducer), by measuring the dimensional and mass changes and the neutralization depths of paste specimens.

In recent years, the use of crushed stone and manufactured sand as concrete aggregates has been increasing, and recycling of the crushed stone powder (CSP) discharged during the production of these aggregates is essential. CSP consists almost entirely of crystals, but it can be used to produce AAMs by using it together with active precursors such as GGBFS [25]. The authors have also confirmed that alkali-activation technology can safely recycle municipal waste incineration bottom ash (BA) [26]. Both CSP and BA are considered to be inactive precursors. To clarify the effect of inactive precursor on the acid resistance of AAMs, this study investigated the acid resistances of GGBFS/CSP-blended and FA/GGBFS/BA-blended AAMs, which are Ca-rich AAMs due to GGBFS addition.

Moreover, in this study, in order to clarify the applicability and limitations of XRD and SEM-EDS, we also discuss the changes in chemical compositions and microstructures that can be detected by XRD and SEM-EDS after Ca-rich AAMs are corroded by sulfuric acid.

## 2. Materials and Methods

### 2.1. Raw Materials 

#### 2.1.1. Precursors

FA and GGBFS were mainly used as precursors of the geopolymers in this study, which met with the JIS (Japanese Industrial Standards) class II (>250 m^2^/kg, Blaine), and the JIS grade 4000, respectively. Table 1 presents their densities, specific surface areas (Blaine values), and chemical compositions. The chemical compositions were determined by X-ray fluorescence spectroscopy (XRF). FA and GGBFS are widely recognized as reactive precursors. The raw stone of the CSP used in this study was hard sandstone. The BA used was discharged from a waste incineration facility in Tottori Prefecture, Japan, in Oct. 2020, with a density of 2.12 g/cm^3^ and a fineness modulus of 3.58. BA usually has a porous structure [26]; correspondingly, its density is smaller than that of natural sand. The chemical compositions and the physical properties of CSP and BA are shown in Table 1. 

#### 2.1.2. Alkali Activator Solution (AS)

Two aqueous solutions of sodium disilicate (called AS10) and caustic soda (called AS01) were first prepared from commercially available sodium silicate, and caustic soda solutions by diluting them with deionized water, respectively. The AS10 has a SiO_2_/Na_2_O molar ratio of 2:1 and a density of 1.352 g/cm^3^, and the AS01 has a concentration of 10 mol/L and a density of 1.334 g/cm^3^. Then, the other three kinds of AS were prepared by blending AS10 and AS01 in a volume ratio of 4:1, 3:1, 2:1, respectively. These three blending AS solutions are referred to as AS41, AS31 and AS21. The physical and chemical characteristics of the five types of AS solution are shown in Table 2. 

#### 2.1.3. Chemical Admixtures

Li et al. developed a retarder (R) and a drying shrinkage-reducing agent (SRA) for geopolymers [27,28]. The former, mainly composed of sodium L-tartrate, can extend the initial setting time of AAFS by 1.8–2.3 times, while the latter, mainly composed of polyether derivatives, can reduce the drying shrinkage strain of AAFS concrete below 400μ. To clarify the effects of chemical admixtures on the acid resistance of AAFS, the retarder, the SRA, and commercial air-entraining (AE) agent for PC concrete were added. The retarder was in powdered form and had a density of 1.82, and the SRA was in liquid form and had a density of 0.93. 

### 2.2. Fabrication of Test Specimens

Table 3 shows the raw materials used and the mix proportions of the AAFS materials. As mentioned above, the BA is porous. Thus, for mixing the AAFS containing the BA, the liquid-filler ratio (AS/F) was set as a large value (0.60). Also, since AAFS has a high viscosity and the unburned carbon in FA absorbs the AE agent, air entrainment is not easy. Though the dosage of AE agent in this study was much larger than the usual value (0.001~0.01% of Portland cement content, by mass) in PC concrete, the AAFS paste sample (series AE) did not have many visible air bubbles. The air content of series AE was not measured due to the lack of an air meter applied to fresh paste. The low flowability of series AE is also considered a possible factor for the low air entrainment. 

The AAFS materials were mixed using a paste mixer in the lab room at 19–23 °C. First, the fillers (and the BA for Series BFSFA (BA)) were placed in the mixer bowl and mixed for 60 s, and then the AS solution was added and mixed for 120 s. The admixtures were dissolved in AS solution and mixed with the fillers. After mixing, prism specimens with the dimensions of 40 mm × 40 mm × 160 mm were produced and then cured in the air at 80 °C for 24 h in a sealed casting surface, followed by unmolding. The unmolded specimens were sealed again and cured in the air at 20 °C. This curing method is called heat-curing here. To investigate the effect of the curing method, some specimens were only cured in the air at 20 °C in a sealed state, referred to as ambient curing here. The specimens used to study the effect of the curing method were cured for up to 28 days, while the other specimens were cured for 7 days.

### 2.3. Acid Resistance Experiments

The sulfuric acid solution used had a pH of 1.0. The pH was checked weekly with a pH meter during the immersion period. The solution was replenished with 64% concentrated sulfuric acid to maintain the pH level by counteracting alkali ions leaching from the specimens.

Before immersing the prism specimens in the sulfuric acid solution, epoxy resin sealing was done to the longitudinal end faces of the specimen. Then, the specimens were immersed in 20 °C water for 2 days to absorb water saturatively. Each specimen’s saturated surface dry weight was measured as its initial mass. The specimens were arranged vertically in the sulfuric acid solution, at intervals of approximately 2 cm, in a box with a cover.

Every 14 days, the specimens were taken out of the sulfuric acid solution and wiped to attain a saturated surface-dry condition. For each specimen, the surface was photographed, and the mass was measured. The cumulative mass change, compared with the initial mass, was determined.

**Figure 1:**
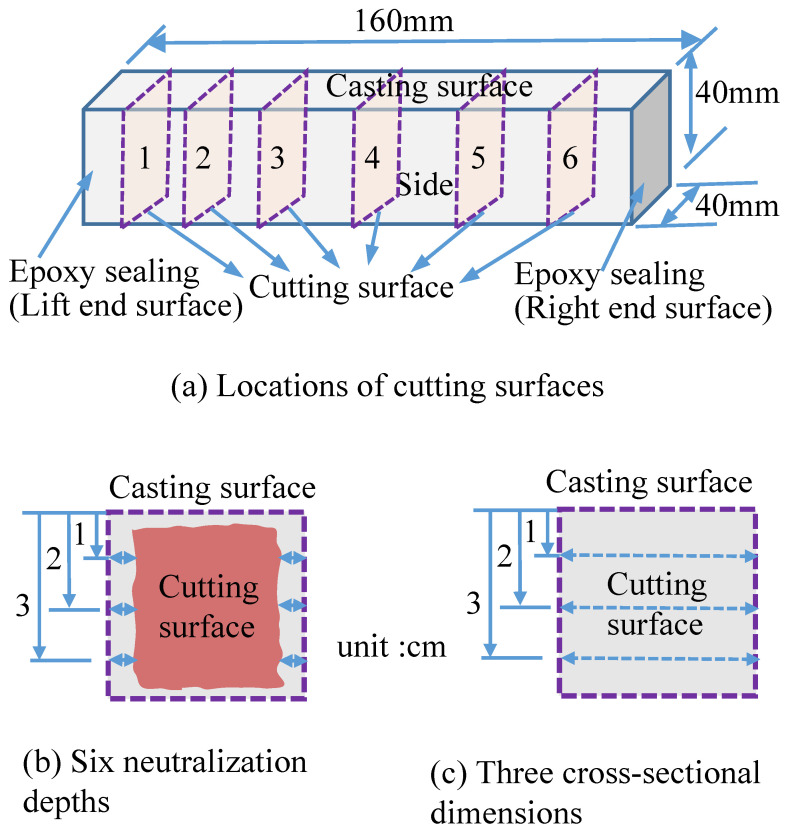
Locations of cutting surfaces (**a**), neutralization depth measurement (**b**), and sectional dimension measurement (**c**).

Then, the specimen was cut without dust-sprinkling measures; the interval of the cutting surface was 20–30 mm, as shown in Figure 1a. A 1% phenolphthalein solution was sprayed on the fresh cutting surface and the depth of colorlessness, called the neutralization depth, was measured, taking an average value of six locations on both sides, as shown in Figure 1b. The distances were also measured at three locations on both sides with an accuracy of 0.01, as shown in Figure 1c. An average value was calculated as the sectional dimension. Six cycles of measurements were performed during the 12-week immersion period.

After each cycle of measurement, epoxy resin sealing was applied to the cutting surface to achieve one-dimensional corrosion, and the mass of the remaining part of the prism was then measured, followed by immersing it in the sulfuric acid solution until the next cycle of measurement.

Recently, Ren & Zhang et al. [29] defined the total degradation depth (TDD) due to acid attack as a sum of the degraded depth (DD), which has been physically removed, and the apparent degraded depth (ADD) where its pH was lowered. As described later in this study, the surface layer of the paste specimens was almost not removed due to acid attack, i.e., the DD was near zero, so the dimensional change (shrinkage or expansion) was used to describe physical degradation, and the neutralization depth, i.e., ADD, represents chemical degradation.

Compressive strength reduction, or residual compressive strength, of a specimen is widely used to identify the effect of acid attack. However, the compressive strength reduction of partially degraded specimens is more significantly affected by the unaffected area than the residual strength capacity of the degraded area. That can be problematic due to possibly increasing strength of the specimen’s unaffected core over time due to continuous hydration and reactions. Therefore, the strengths before and after sulfuric acid immersion were not measured and compared in this study.

### 2.4. XRD and SEM-EDS Analysis

XRD and SEM/EDS analysis were carried out for three series: AS21-45, BFSCSP, and BFSFA(BA), cured by the heat-curing method. After 84-day acid immersion, the specimens were taken out of the acid solution, then the analysis samples were prepared. The material ages of the specimens when they were analyzed were about three months. The operational conditions of the XRD analysis were as follows: 40 kV–15 mA X-ray tube power, doubly Ni-filtered CuKα radiation, 4°/min–0.01° step scan, 1.25°–10 mm–13 mm–13 mm slit system, and 5–70°, 2θ range. The SEM-EDS analysis was taken under 15 kV accelerating voltage. ZAF (atomic number, absorption and fluorescence) corrections were automatically done for point analysis data. The primary data obtained by the EDS analysis were in atomic percentage. Besides Ca/Si and Al/Si atomic ratios, the CaO and SO_3_ mole percentages were calculated for discussing the changes in the gel compositions of AAFS paste after exposure to the sulfuric acid solution.

## 3. Results and Discussion

### 3.1. Effects of Curing Methods on the Acid Resistance of AAFS

**Figure 2:**
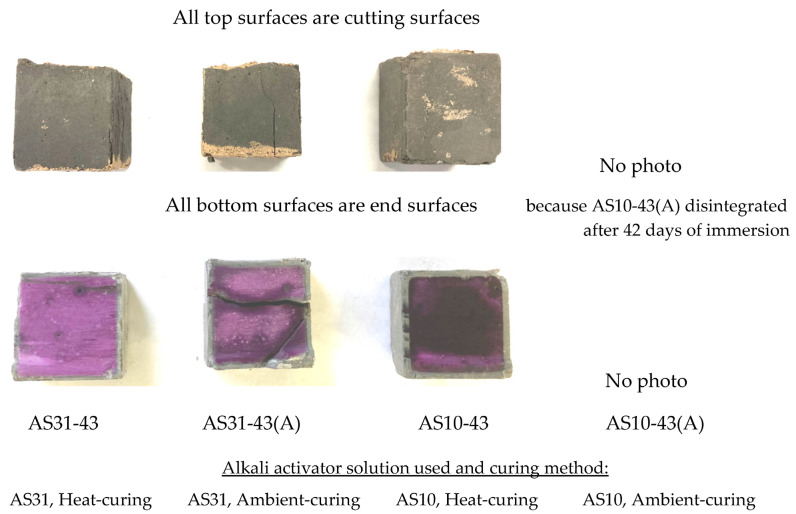
Appearance (upper row) and coloring (lower row) of the specimens using different AS and cured by different methods after 84-day immersion (Clay color in the specimens’ surfaces is due to epoxy sealing or its contamination).

Figure 2 shows the degradation situations of the four series of specimens with different curing methods and AS solutions. The AS10-43(A) cured by the ambient-curing method was broken at 42 days of acid immersion. Compared with the heat-cured specimens, the AAFS binders cured only in the ambient air had lower acid resistance. This was attributed to a more stable cross-linked aluminosilicate structure formed in the heat-cured specimens [21]. The higher permeability of the ambient-cured specimens is another reason. Heat curing promotes the polymerization reaction, resulting in a dense internal structure. 

Since only sodium silicate was used as an alkali activator, the denseness and strength of the AS10-43(A) specimen were low, so sulfuric acid might have penetrated and caused the specimen to deteriorate severely and disintegrate. Both AS31-43 and AS10-43 cured by the heat-curing method had cracks, but it seems that the surface deterioration and cracks of AS10-43 were more obvious. As described in Section 3.3, AS with a suitable blend of sodium silicate and caustic soda can yield a more stable binder than if used alone. 

**Figure 3:**
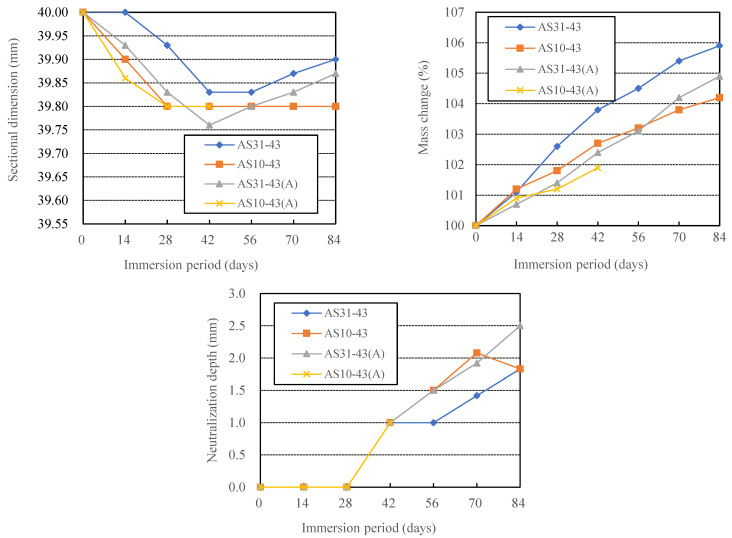
Effects of curing methods on the acid resistance of AAFS.

Figure 3 shows the variation of sectional dimension, mass change, and neutralization depth with immersion period for the four series of AAFS paste specimens. The results of past acid resistance experiments by other researchers reported either a gradual decrease in the sample dimensions due to spalling of the corroded zone or a gradual dimensional increase due to the generation of expansive gypsum crystals [14,30]. However, we found in this study that the size of the AAFS specimens first decreased and then increased or remained constant until 84 days.

The corrosion mechanism of hardened AAFS paste in sulfuric acid (pH ≒ 1.0) consists of two steps [31,32]. The first step of acid attack is an ion exchange reaction between the charge-compensating cations (Na^+^, Ca^2+^) of C-N-A-S-H gels and the H^+^ or H_3_O^+^ ions from the acid solution with an electrophilic attack by acid protons on polymeric Si−O−Al bonds. The Ca ion separation from C-N-A-S-H gels is called decalcification. The electrophilic attack results in the ejection of tetrahedral aluminum from the aluminosilicate framework, so-called dealumination. Decalcification and dealumination increase the porosity of the geopolymer structure and may cause shrinkage cracks. In the second step, the exchanged Ca^2+^ ions diffusing toward the acid solution react with counter-diffusing sulfate anions, resulting in the formation and deposition of gypsum crystals inside the corroded area. According to this corrosion mechanism, the depolymerization caused the dimensional decrease of the specimen in a certain period because there was not much gypsum formation in this period. Of course, very slight surface spalling was also responsible for the size reduction, although photography does not detect minor spalling easily. In summary, gel shrinkage and surface spalling resulted in a dimensional decrease. Once the gypsum was formed in large amounts, the dimension of the specimens changed from decreasing to increasing. The specimens without aggregates could deform freely, which led to the detection of dimension change in this study. Gu et al. [33] reported that AAF concrete, immersed in 1% sulfuric acid solution and subjected to a 12 h wetting and drying cycle, showed a dimensional decrease up to about 50 days, followed by an increase. The geopolymer mortar using fine lignite bottom ash also found a length decrease up to about 60 days, then a length increase after immersion in 5% Na_2_SO_4_ solution [34]. 

As shown in Figure 3, the ambient-cured specimens had a larger dimensional decrease and increase than those cured by the heat-curing method (see AS31-43 and AS31-43(A)). Thus, the degradation of AS31-43 was more severe than that of AS31-43(A), as shown in Figure 2. Moreover, compared with the other three specimens, the AS10-43(A) specimen had the largest dimensional decrease rate. However, AS10-43 showed almost no dimensional change after 28-day immersion. As described in Section 3.3, we found that the smaller the blending ratio of caustic soda in the AS solution, the less the expansion of the AAFS specimen. Although the sectional dimensions of the specimens shifted from decreasing to increasing in the immersion period, the dimensions of the specimens after the 84-day immersion were smaller than their respective initial dimensions.

On the other hand, all specimens’ mass and neutralization depth increased in the immersion period. As shown in Figure 2, there was no visible localized spalling on the surface of the three series of specimens, except for cracks. As mentioned above, Lee and Lee [19] observed a mass gain when the AAFS binder contained 50% slag, while less than 30% blending ratio of slag yielded mass loss. The internal diffusion of sulfuric acid and the formation of gypsum are considered to contribute to the mass increase. However, in the case of AAFS mortar, mass loss was found when slag was 0%, 20%, 40% and 100%, while the AAFS mortar using 70% slag almost had no mass change [17]. The surface deterioration, corroded zone spalling, and gypsum crystals’ formation determines the mass change simultaneously. The surface deterioration is predominantly caused by the combined decalcification and dealumination of calcium sodium aluminosilicate hydrate (C-N-A-S-H) gels [17]. The constraint of aggregate on the shrinkage or expansion of the binder matrix may enhance the surface deterioration and the spalling. The present result showed that 40% slag blending ratio led to a mass gain of AAFS binder without aggregate addition. The mass increases of AS10-43 and AS31-43(A) were near, but AS31-43 had a large mass increase. Though the mass increase of AS31-43(A) was smaller than that of AS31-43, the former had a larger dimensional increase after 42-day immersion. This suggested that there is no inevitable correlation between mass change, dimensional change, and appearance damage that is called physical degradation of AAMs, such as roughening, scaling, spalling, popping, and cracking of the corroded zone. The dimensional change depends not only on the amount of gypsum produced but also on the degree of the denseness of the AAMs. Whether the physical degradation occurs or not is determined by the degree of dimensional change and the strength of the AAMs, as well as the constraint of aggregate on the dimensional change of the matrix.

The AAFS binder, which has not been subject to acid attack, generally has high alkalinity (i.e., ranging from 10.5 to 12.0 [35]) due to residual alkali activator solution in the pores. The acid penetration through the pores and the leaching of alkalis towards the exterior reduces the internal alkalinity, i.e., neutralization. Like Portland cement, even if pH decreases below 10.0, the neutralization may not be a problem for the alkali-activated binder itself, but rebar corrosion may occur. The neutralization rate depends on the initial alkalinity of the binder and the rate of acid penetration. The influencing factors and consequences of neutralization are clearly different from the physical degradation. 

Due to the different mechanisms of physical degradation and neutralization, we proposed distinguishing acid corrosion of AAMs as physical and chemical degradation to discuss the influencing factors of acid resistance rationally. The physical degradation is manifested by appearance damage caused by shrinkage or expansion and size reduction when spalling occurs. The neutralization was referred to as chemical degradation here. A pH indicator, such as phenolphthalein solution, can be used to identify the neutralization depth (ND) after an acid attack [30]. 

As shown in Figure 2 (lower row), the acid-exposed samples exhibited a light pink-colored core and a colorless outer layer. The coloring pH of the phenolphthalein solution was above 10.0. Thus, the coloring does not mean that the pH was not reduced from its initial value. Therefore, in this study, the non-chemically degraded zone, judged by the color change after spraying the pH indicator, only indicates that the zone had not yet suffered so serious chemical degradation that the pH decreased below 10.0, above which the passive reinforcing steel is generally considered to remain without starting to rust. As shown in Figure 3, AS31-43 had the minimum ND. The NDs of AS31-43(A) and AS10-43 were roughly the same. This means that heat curing and the addition of sodium hydroxide can reduce the chemical degradation of AAFS.

### 3.2. Effects of Alkali Activator Solution-Filler Ratio

**Figure 4:**
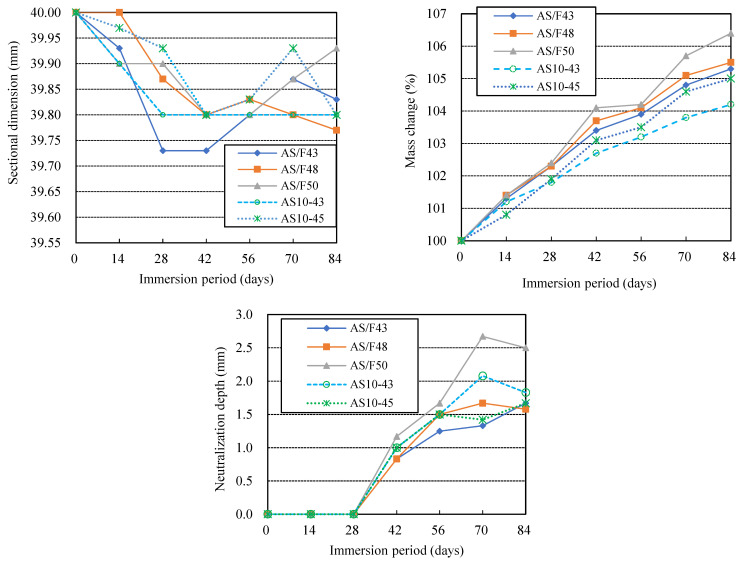
Effects of alkali activation solution-filler ratio (AS/F) on the acid resistance of AAFS.

**Figure 5:**
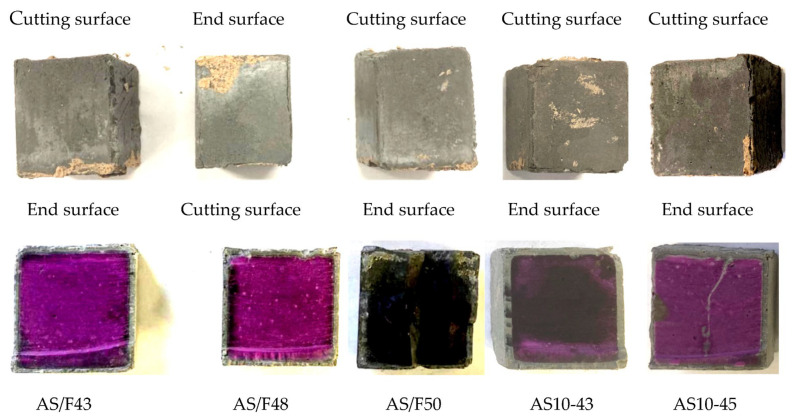
Appearance (upper row) and coloring (lower row) of the specimens with different AS/F after 84-day immersion.

Figure 4 shows the changes in sectional dimension, mass, and neutralization depth with immersion time for AAFS with different liquid-filler ratios (AS/F). As the liquid-filler ratio decreased, the dimensional reduction rate increased at the initial stage. Still, no certain pattern was found for the effect of the liquid-filler ratio on dimensional recovery or increase. The dimensional recovery or increase depends not only on the number of expansive substances formed but also on the denseness of the specimen. However, as shown in Figure 5, compared with AS/F43 and AS10-43, the other three series (AS/F48, AS/F50, and AS10-45), which had larger AS/F, presented obvious cracks and severe physical degradation, especially cracks found inside the AS/F50 and AS10-45 specimens. It should be noted that the fabrication of the specimen left the right corners and surface defects of AS/F43. Therefore, besides the acid attack intensity, the physical degradation also greatly depends on the strength and denseness of the material, and the aggregate’s constraint on the volumetric change of the matrix binder. The results of this study suggested that the larger the liquid-filler ratio, the more severe the physical degradation. 

The mass increase of AAFS specimens immersed in sulfuric acid should be attributed to the absorption of sulfuric acid and gypsum formation. As shown in Figure 5, the larger the liquid-filler ratio, the greater the mass increase. The larger the liquid-filler ratio, as with hardened PC pastes, the more numerous the pores in the AAFS specimen and the corresponding increase in sulfuric acid absorption. As shown in Figure 5 (lower row), The core of the AS/F50 specimen exhibited a dark color, which is thought to be due to internal wetting, indicating that the AS/F50 specimen with larger AS/F had higher water permeability. 

In the case of using a blend of sodium silicate and sodium hydroxide as AS solution (AS31), the ND increase also showed the same pattern as the mass increase. The larger the liquid-filler ratio, the greater the neutralization depth, i.e., the more severe the chemical degradation, though the ND difference between the specimens was very small. Obviously, the AS/F50 showed severe neutralization. The liquid-filler ratio yields an uncompacted microstructure of AAM, which allows acid ingress and causes the easy release of Na or K ions [36]. However, for AS10-43 and AS10-45, which used only sodium silicate as AS solution, the slight change in the liquid-filler ratio brought little difference in the neutralization depth. This is because easy neutralization caused by the initial low alkalinity made the effect of AS/F insignificant.

### 3.3. Effects of AS Components

**Figure 6:**
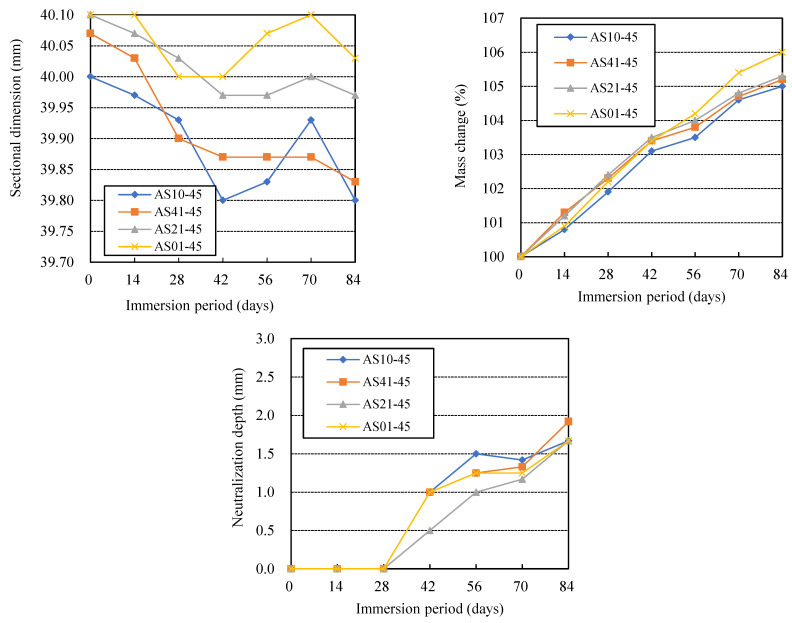
Effects of alkali activation solution’s components on the acid resistance of AAFS.

**Figure 7:**
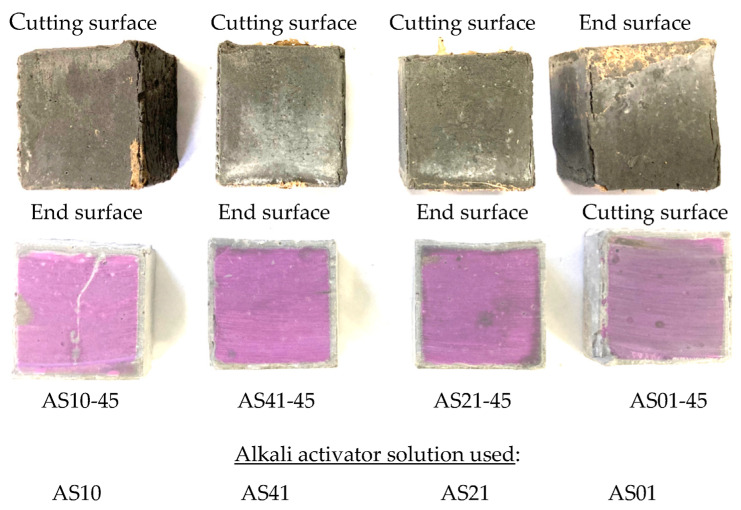
Appearance (upper row) and coloring (lower row) of the specimens using different AS solutions after 84-day sulfuric acid immersion.

From the dimensional changes of the specimens in the immersion period shown in Figure 6, it can be found that AS10-45 and AS41-45 using AS10 and AS41 as AS solution exhibited a large dimensional decrease in the early stage of immersion but almost no dimensional increase in the later stage of immersion. However, AS01-45 using only sodium hydroxide as AS solution showed a small dimensional decrease in the early immersion stage and almost recovered to its initial value in the later stage. By comparing the results of the four series of AAFS specimens, we found that the larger the blending ratio of sodium silicate in the AS solution, the greater the dimensional decrease, and the smaller the dimensional increase of AAFS binder after the dimensional decrease. Large dimensional changes caused significant cracks and surface deterioration in AS10-45 and AS01-45, as shown in Figure 7. Carefully comparing the widths of the cracks at the edges of AS41-45 and AS21-45, it can be found that the physical degradation of AS21-45 was milder. That is to say, the higher the content of sodium silicate in the blended AS solution, the more likely the AAFS binder was to undergo physical degradation, and the AAFS binders using a sodium silicate or sodium hydroxide solution more easily suffer physical degradation than those using a blend of them. The authors found that the strength of AAFS concrete, using a blended AS of NaOH and sodium silicate, decreased as the fraction of sodium silicate in the AS increased [37]. Greater strength should make AAMs less susceptible to physical degradation, such as cracking. 

As indicated in Figure 6, the greater the fraction of sodium silicate in the AS solution, the greater the mass increase, which agreed with the degree of dimensional increase of each specimen in the later immersion period. The AA10-45 and the AA41-45, which had a larger blending ratio of sodium silicate in the AS solution, had a larger neutralization depth due to lower initial alkalinity than the AA21-45. Thokchom et al. also reported that AAF mortar with lower content of Na_2_O in a blended AS solution of sodium hydroxide and sodium silicate, i.e., larger content of sodium silicate, had a faster neutralization rate during 10% sulfuric acid immersion [22]. However, the AS01-45, which used only sodium hydroxide as AS, had a greater neutralization depth than the AS21-45. Therefore, the AAFS using a blended AS had a higher resistance to chemical degradation, especially when the blended AS had a higher sodium hydroxide content.

### 3.4. Effects of Precursor

**Figure 8:**
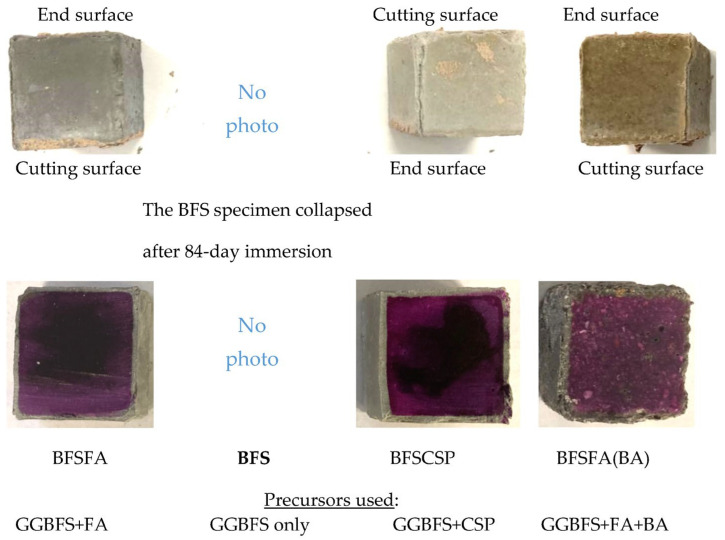
Appearance (upper row) and coloring (lower row) of the specimens using different precursors after 84-day sulfuric acid immersion.

As shown in Figure 8, cracks appeared in the edges of all three series of specimens using 50% GGBFS, and the BFS specimens using only GGBFS as fillers even broke. Also, comparing the surface deterioration of the three specimens AS31-43 (see Figure 2), AS/F43 (see Figure 5), and BFSFA, we found that the cracking of BFSFA was the most serious. The BFSFA used 50% GGBFS, while the AS31-43 and the AS/F43 used 40% GGBFS. Considering that the BFS specimens were broken, it can be concluded that if the blending ratio of GGBFS in the fillers is over 50%, the physical degradation resistance of AAFS to sulfuric acid becomes low, as reported by Lloyd et al. [18] and Lee et al. [19]. 

**Figure 9:**
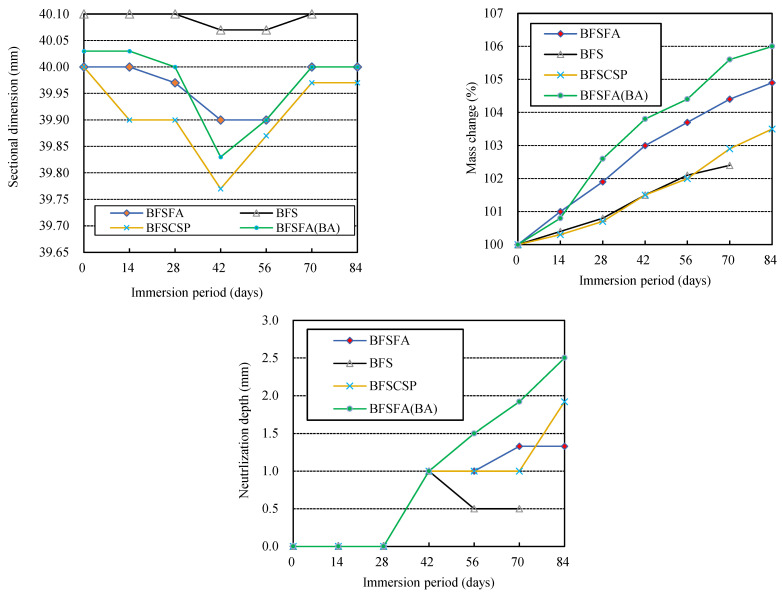
Effects of precursor used on the acid resistance of geopolymer.

We also observed from Figure 8 that the BFSCSP specimen using CSP to replace the FA, and the BFSFA(BA) specimen, in which the urban waste incineration bottom ash (BA) was added, had larger cracks than the BFSFA specimen. The BFSCSP specimen and the BFSFA(BA) specimen had large shrinkage and expansion rates, as shown in Figure 9. Though the BFS specimen (AAS) using only GGBFS as fillers had a small dimensional decrease (shrinkage) and expansion, it was broken down because it was dense, compared with the BFSFA specimen (AAFS). The small mass increase and the small neutralization depth of the BFS specimen were due to its higher denseness. Therefore, visible physical degradation of Ca-rich AAM is caused by various factors and does not depend only on the number of corrosion products. At present, we do not know why the BFSCSP specimen had a smaller mass increase than the BFSFA specimen. This may be because FA has a small amount of calcium, as well as a higher water absorption capacity than the CSP. 

The CSP and the BA are inactive, and the BA has a porous structure, so the polymerization reaction products in the BFSCSP specimen should be less than that of the BFSFA specimen, and the permeability of the BFSFA(BA) should be greater than that of the BFSFA. Permeable voids and high water absorption of AAM can contribute to the H_2_SO_4_ attack of AAFS [19]. Thus, the neutralization depths of the two specimens were greater than that of the BFSFA. The BFSFA(BA) chemical degradation was the fastest in the sulfuric acid solution among the four specimens. Hence, replacing FA in AAFS with CSP or adding BA to AAFS will increase Ca-rich AAMs’ physical and chemical degradation. Li et al. confirmed that AAFS mortar using the BA has lower carbonation resistance, compared to the use of sea sand, due to the porous feature of BA particles [26,38]. 

### 3.5. Effects of Chemical Admixture Addition

**Figure 10:**
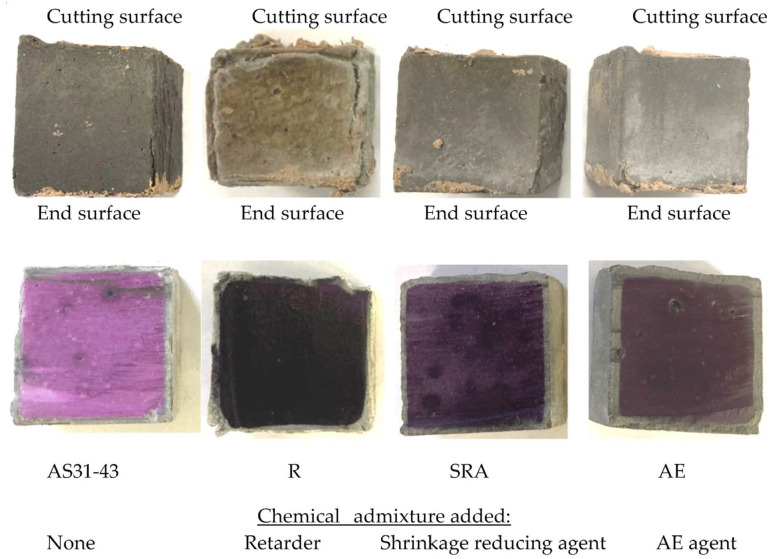
Appearance (upper row) and coloring (upper row) of the specimens (AS/F = 0.40) using different chemical admixtures after 84-day sulfuric acid immersion.

Figure 10 shows the appearance and coloring of the specimens using different chemical admixtures after 84-day sulfuric acid immersion. The physical degradation of the R specimen with retarder addition was very severe. Still, the SRA specimen with the shrinkage-reducing agent and the AE specimen with the air-entraining (AE) agent showed very slight surface deterioration, even lower than that of the AS31-43 specimen without adding any chemical admixture. 

**Figure 11:**
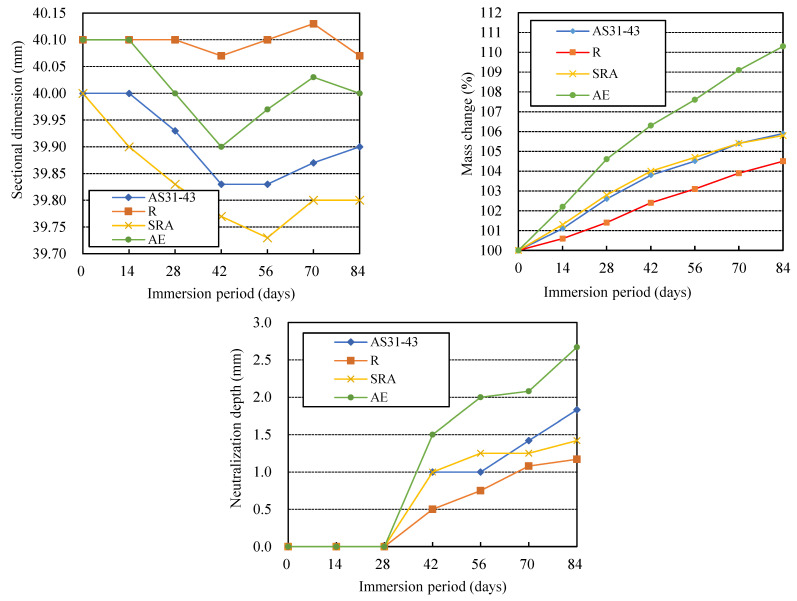
Effects of chemical admixture on the acid resistance of AAFS.

Sodium tartrate, being the main component of the retarder, combines with the Ca^2+^ ions dissolved from GGBFS at the initial stage to form the chelate compounds, which cover the surfaces of GGBFS particles to retard the further dissolution of Ca^2+^ ions. Thus, sodium tartrate can act as a retarder of AAMs using GGBFS [39]. This chelate compound may be susceptible to acid attack, so the R specimen was severely degraded. However, the mass increase and dimensional change of the R specimen were very small, as shown in Figure 11.

The AE specimen showed a mass increase of more than 10%, but the dimensional decrease was insignificant and did not yield a dramatic expansion. The tiny voids introduced by the AE agent were believed to moderate the internal stress caused by shrinkage or expansion. Therefore, little surface deterioration was observed in the AE specimen. However, the AE specimen’s neutralization depth was large due to the great penetration of sulfuric acid promoted by the tiny voids. Therefore, the chemical degradation of AAFS with AE agent should be a concern. 

There is almost no difference in the chemical degradation between the SRA specimen with the dry shrinkage reducer (SRA) and the AS31-43 specimen without adding any admixture from the neutralization depth. The mass increases were also almost the same. Although the dimensional decrease of the former was slightly greater than that of the latter with a slightly larger liquid-filler ratio, the surface degradation of the former was milder. Thus, it can be concluded that the addition of SRA has almost no effect on the acid resistance of AAFS. It must be noted that, although the colorless zone of the R specimen was shallow, i.e., the measured neutralization depth was small, its colored core area appeared black. We suspect sulfuric acid entered the core area via fine cracks, and the chemical degradation in the black-colored core area also occurred. 

### 3.6. XRD Analysis

XRD analysis was performed for three series of specimens, AS21-45, BFSCSP, and BFSFA(BA) to identify the changes of crystalline compositions after the sulfuric acid immersion. XRD samples were taken from the outer area and the inner area of each of the specimens. The former was colorless, whereas the latter exhibited pink after spraying the phenolphthalein solution. 

**Figure 12:**
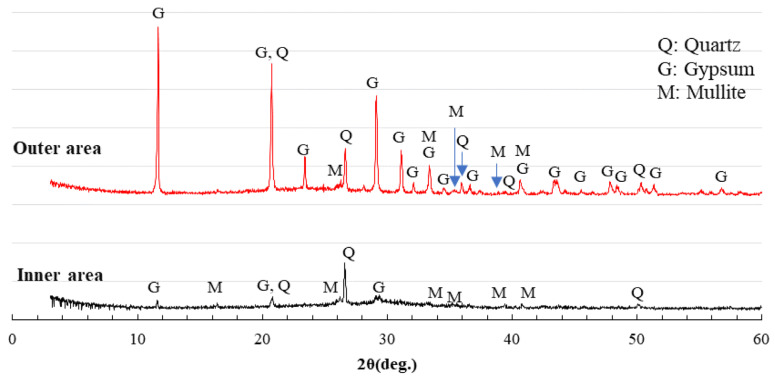
XRD patterns of series AS21-45 after sulfuric acid immersion.

Figure 12 shows the XRD patterns of AS21-45. The mullite and quartz in the outer and inner areas originated from the fly ash. The raw GGBFS had no gypsum addition, but in the outer area, gypsum was found, i.e., the decalcification reaction occurred, which refers to the dissolution of Ca ions from C-N-A-S-H gels and further the formation of gypsum crystals with sulfuric acid. However, a very small amount of gypsum was also found in the inner area of AS21-45. A trace amount of gypsum was generated due to the internal diffusion of sulfate ions. Though the formation and deposition of gypsum crystals may help resist further acid penetration and attack, inhibiting the total process of deterioration [31], calcium sulfate may also form on the inward side of the chemical degradation zone. However, since less gypsum was formed inside the AS21-45 specimen, its physical degradation was very light, as shown in Figure 7. Although sulfuric acid had penetrated into the non-chemically degraded area with gypsum formation, the inner area still had color after spraying the phenolphthalein solution because of the limited external leaching of Na^+^, K^+^ ions.

**Figure 13:**
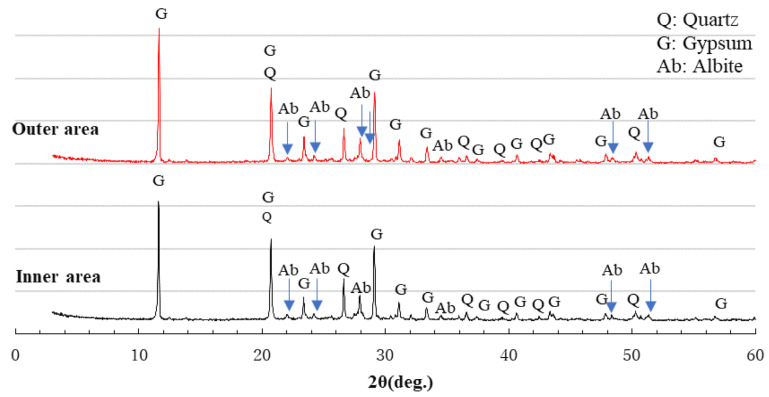
XRD patterns of series BFSCSP after sulfuric acid immersion.

Figure 13 shows the XRD patterns of the BFSCSP specimen. The hard sandstone CSP used contains quartz, albite, calcite, anorthite, vermiculite, etc. These crystals should not be involved in the polymerization reaction and were found in the BFSCSP specimen before the sulfuric acid immersion [25]. However, in the XRD patterns of the BFSCSP specimen after exposure to the sulfuric acid solution, the calcite, anorthite, and vermiculite peaks disappeared, and only the peaks of quartz and albite were found. The three absent crystalline compounds might have been dissolved by sulfuric acid attack. In addition, almost the same gypsum formation was found in the outer and inner areas. The severe cracking of the BFSCSP specimen, as shown in Figure 8, should be due to the expansion stress caused by the formation of much gypsum in the inner area. 

Since crystalline CSP is not involved in the polymerization reaction, the reaction products of the BFSCSP specimen should be similar to those of alkali-activated slag (AAS). As mentioned before, the acid resistance of AAS is lower than that of AAFS. The colored inner area of the BFSCSP specimen, determined with the phenolphthalein solution, showed dark black rather than red or purple, as shown in Figure 8, which implies that sulfuric acid has penetrated inside and thus led to the gypsum formation. 

**Figure 14:**
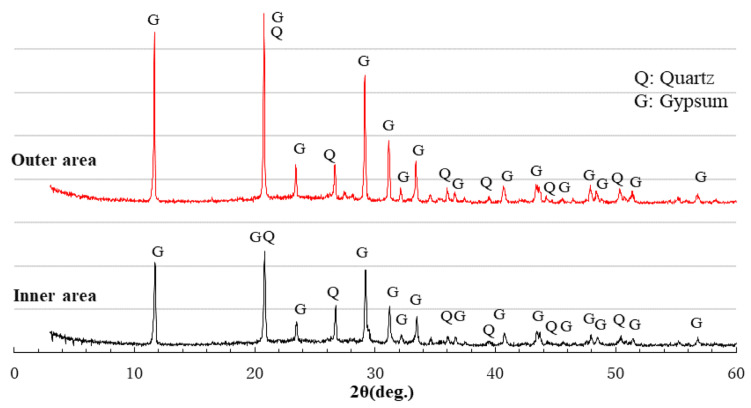
XRD patterns of series BFSFA(BA) after sulfuric acid immersion.

Figure 14 shows the XRD patterns of the BFSFA(BA) specimen. In other studies [26], the authors found that raw BA has crystalline components of quartz, albite, calcite, katoite, hematite, potassium magnesium hydride, and carbonated sulfuric acid Afm (Ca_4_Al_2_O_6_(CO_3_)_0.67_(SO_3_)_0.33_·11H_2_O, here referring to as C4. These crystals, except the C4, were found in the AAMs using GGBFS, FA, and BA. The AS solutions dissolved the C4, and were further converted to gobbinsite. However, in this study, only quartz, mullite and gypsum were observed in the XRD patterns of the BFSFA(BA) specimen after being immersed in the sulfuric acid solution. The FA used contains quartz and mullite. Albite was not detected, most likely because the XRD samples contained few BA. Since BA is not powder, the collection location of the XRD samples affected the XRD results. However, there were gypsum crystals in the outer and inner areas, although the gypsum peaks in the inner area were lower than those in the outer area. The large amount of gypsum formed inside the BFSFA(BA) specimen was responsible for its considerable physical degradation.

Comparing the heights of the gypsum peaks of the XRD patterns of the inner area in Figure 12 and Figure 14, it can be seen that more gypsum was formed in the inner area of the BFSFA(BA) specimen than in the AS21-45 specimen. This indicates that the incorporation of porous BA increased the penetration of sulfuric acid and decreased the acid resistance of AAFS. Similarly, by comparing Figure 12 and Figure 13, it can be found that the acid resistance of the GGBFS/CSP blend-based binder is lower than that of AAFS.

XRD analysis can undoubtedly identify whether gypsum was formed in the AAM after sulfuric acid immersion. XRD analysis without the addition of standard substances can only be used to determine the type of crystalline compounds. However, cross-reference to Figure 7, Figure 8, Figure 12, Figure 13 and Figure 14 reveals that the higher the gypsum peak in the XRD pattern of the inner area, the more severe the physical degradation of the AAM. Therefore, comparing gypsum peak intensities of inner areas would reveal the differences in acid resistance of different Ca-rich alkali-activated binders.

### 3.7. SEM-EDS Analysis

**Figure 15:**
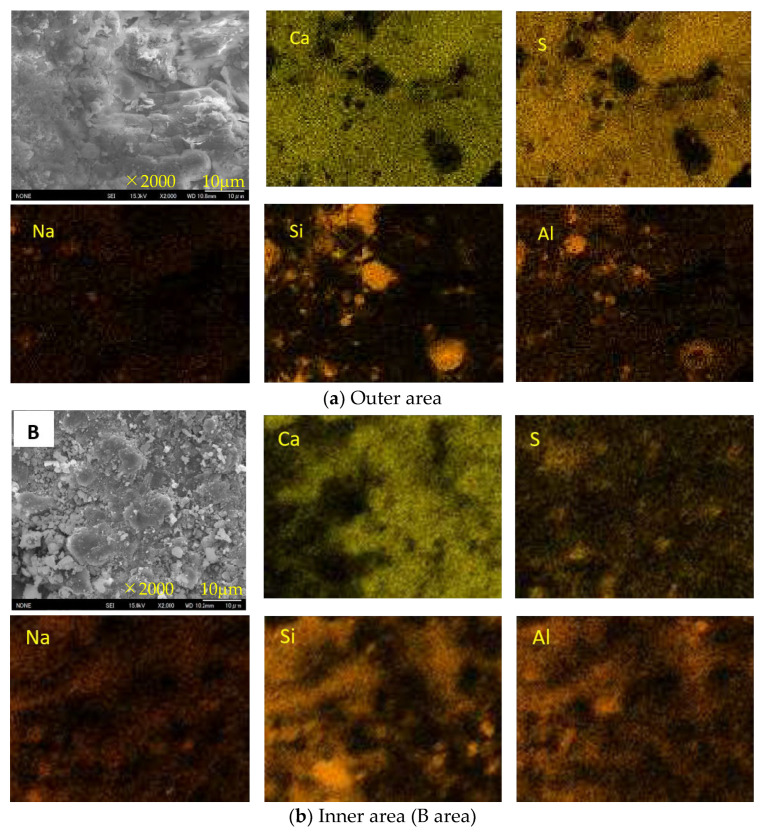
SEM images and element maps of the AS21-45 specimen.

**Figure 16:**
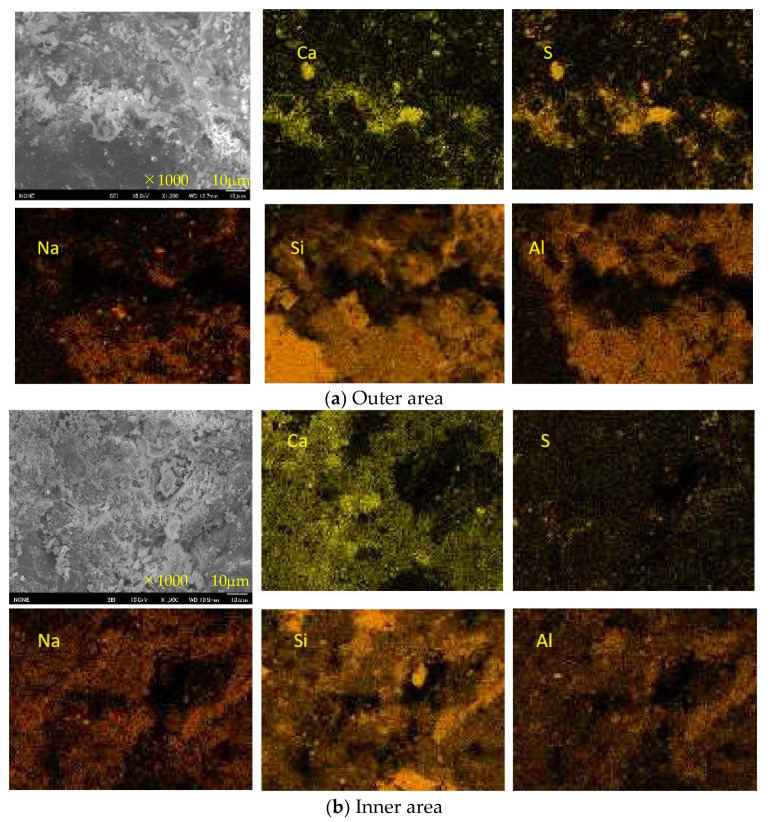
SEM images and element maps of the BFSCSP specimen.

**Figure 17:**
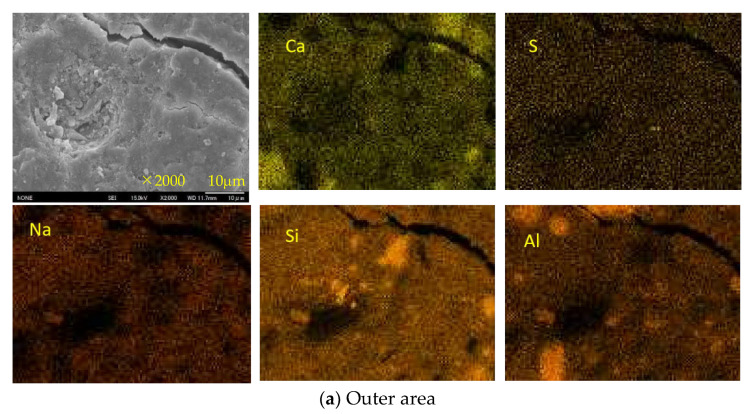

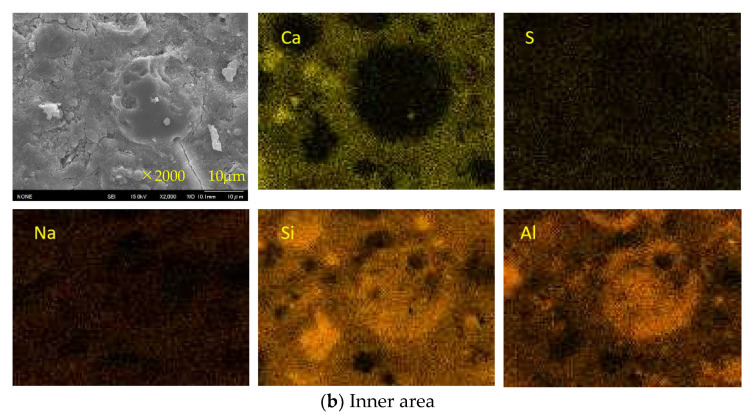
SEM images and element maps of the BFSFA(BA) specimen.

Figure 15, Figure 16 and Figure 17 show the SEM images and element maps of the outer and inner areas of the AS21-45, BFSCSP, and BFSFA(BA) specimens after being exposed to the sulfuric acid solution for 84 days. As shown in Figure 15 and Figure 16, a large amount of gypsum crystals were found in the outer area of the AS21-45 specimen and the BFS/CSP specimen. Still, according to the S-element maps, only a small amount of gypsum deposits were formed in the inner area. The inside Ca and S element distributions suggest that almost all the Ca were still retained in the geopolymeric gels, and only a few Ca were bound to sulfur inside the two specimens. From the Na element maps, it was found that Na in the outer areas decreased, compared to the inner areas, suggesting Na^+^ leached out of the frameworks of two binders, so-called dealkalization. However, Si and Al elements had almost the same distribution, even in the outer area. Therefore, the tetrahedral aluminum remains in the original structural system of C-A-S-H gel, even if dealumination occurs.

However, we did not observe much gypsum in the SEM images of the outer area of the BFSFA (BA) specimen from Figure 17, although XRD analysis detected many gypsum crystals. However, a careful comparison of the outer and inner S-element maps shows that dotted gypsum crystals were generated and dispersed in the outer area. The collection position of the sample for SEM analysis inevitably affects the analytical results. Another speculation is that BA is porous, which provides an easy way for sulfuric acid penetration and easily accommodates ions, so that gypsum might precipitate within the BA particles. Since the BA particles were wrapped within the geopolymeric gels, the interiors of the BA particles cannot be easily observed using a non-polished SEM sample. Compared to the AS21-45, the Si and Al elemental maps of the BFSFA(BA) are less correlated, and the inside Na content is less than that of the outside. The inhomogeneity of the chemical components of BA particles and the inhomogeneous distribution of BA particles in the AAM caused the difficulty in interpreting the elemental maps of the BFSFA(BA). 

Decalcification, dealumination, and dealkalization of geopolymer gels are generally considered to be the acid corrosion mechanism of AAMs. Therefore, for homogeneous Ca-rich alkali-activated binder subjected to acid attack, SEM images and Ca, S, Na-element maps of SEM-EDS analysis can be used to determine the occurrence of decalcification and dealkalization of C-N-A-S-H gels, but it is difficult to judge the dealumination in the gel structure system.

The variations of Ca/Si and Al/Si atomic ratios from SEM-EDS analysis have often been used to evaluate the acid attack of AAMs. Komljenović et al. [40] calculated the average Ca/Si and Al/Si atomic ratios in the AAS specimen after immersion in a sulfuric acid solution and found that the Ca/Si atomic ratio and Na atomic percentage decreased with increasing sulfuric acid immersion time. Still, the Al/Si atomic ratio increased slightly. The Ca/Si ratio decreases due to decalcification and calcium sulfate formation, and the decrease in Na is due to the dissolution of the geopolymeric gels into the sulfuric acid solution. Gu et al. [33] performed a surface elemental mapping analysis for the AAF subjected to sulfuric acid attack. They reported that the Al/Si atomic ratio decreases due to sulfuric acid immersion. However, although Ca, Al, and Na are separated from the C-A-S-H gel structure, Al and Na may remain within the gel framework. The regional EDS analysis may not necessarily reflect the compositional changes of the C-N-A-S-H gels. Therefore, SEM-EDS point analysis was also performed on the AAFS specimen in this study. However, since the Ca, Al, Si, and Na components in the inactive CSP and BA are almost unrelated to the gel formation of AAMs, they would confuse the percentages of Ca, Al, Si and Na atoms and the interpretation of the changes in atomic ratios. Therefore, the EDS point analysis of this study was only for the AS21-45 specimen.

**Figure 18:**
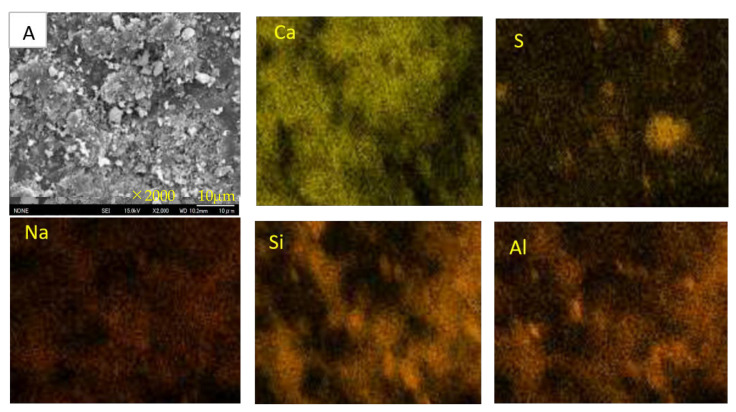
SEM images and element maps of the AS21-45 specimen (inner area A).

Figure 18 shows an SEM-EDS analysis for another inner area A in the AS21-45 specimen. The atomic percentage of Si, Na, Al, Ca in this area, Ca/Si and Al/Si atomic ratios, as well as the CaO/SO_3_ mole ratio, will be compared with other areas and points, as explained later. From the S-map, it was found there was not much sulfuric acid entering in area A, compared to area B. The Na, Al, and Si elemental maps show similar patterns, suggesting that there was no significant dealkalization and the dealumination of C-A-S-H gels.

**Figure 19:**
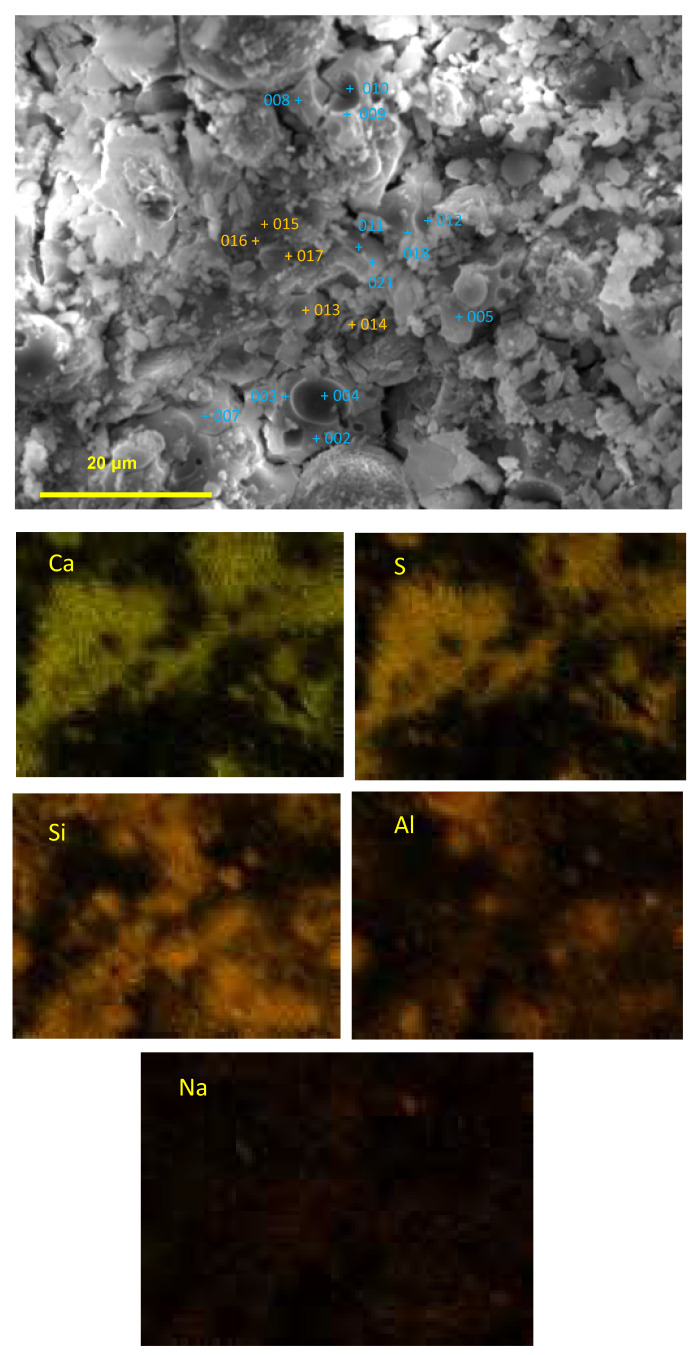
Point analysis and elemental mapping of the outer area of the AS21-45 specimens after sulfuric acid immersion.

**Figure 20:**
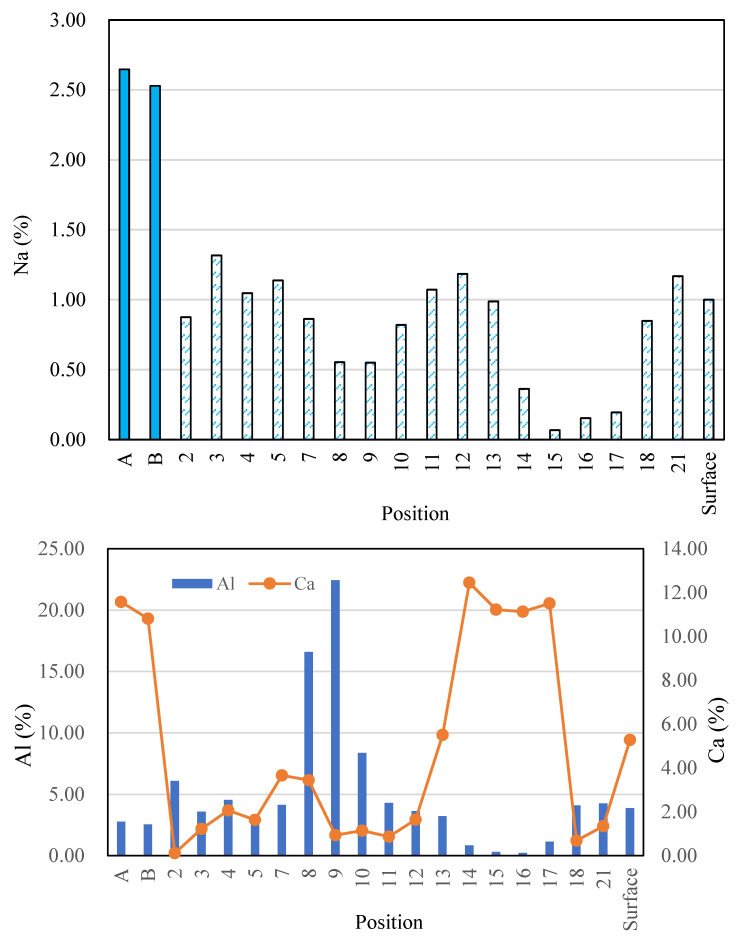
Na, Al, Ca atomic percentages at the outer and inner areas (A, B: average values of each inner area, see Figure 15b and Figure 18; Numbers: analysis results of the points in the outer area; and Surface: average value of the outer area, see Figure 19).

Point analyses were conducted for 17 points at the outer area of the AS21-45 specimen, as shown in Figure 19. The Ca and S maps have a similar patten, but the Ca, Na, Al, and Si elemental maps are different from each other, which indicates that gypsum formation, dealkalization, decalcification and the dealumination of C-A-S-H gels occurred in the outer area. Figure 20 shows the Na, Al, and Ca atomic percentages (%) in the inner areas A and B (see Figure 15 and Figure 18), the outer (surface) area, and the 17 points on the outer area. Due to the minor acid attack in the inner areas A and B, the atomic percentages of these two areas can be used as an approximate reference for assessing the elemental changes of each point in the outer area. The Na atomic percentages of the outer area (“surface”) and all the 17 points in the outer area were smaller than those of the inner areas A and B, but the same pattern was not found for the Al and Ca elements of the 17 points. The Ca in the outer area (“surface”) was less than that in the inner areas A and B, but the outer area (“surface”) had more Al than the inner areas A and B. In other words, the atomic percentage of Na at each point can indicate the dealkalization behavior after an acid attack, but it cannot be estimated from the atomic percentages of Al, Ca whether dealumination and decalcification have occurred at each point.

**Figure 21:**
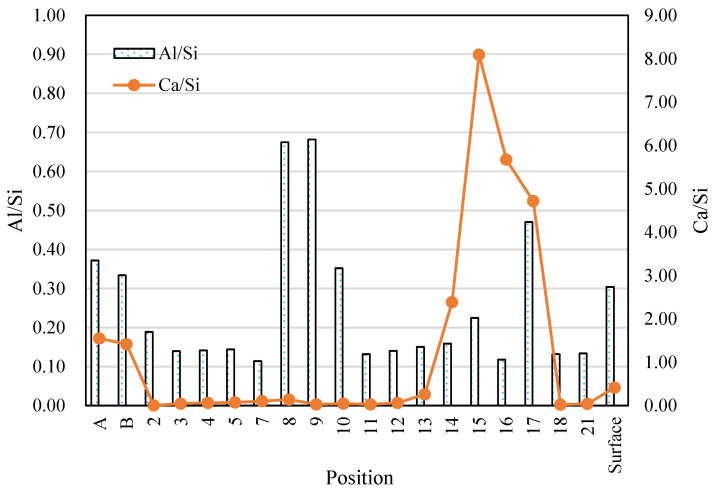
Atomic ratios at the outer and inner areas (A, B: average values of each inner area, see Figure 15b and Figure 18; Numbers: analysis results of the points in the outer area; and Surface: average value of the outer area, see Figure 19).

As shown in Figure 21, the atomic ratios of Al/Si and Ca/Si at each point of the outer area were different, and some were larger than those in the inner areas A and B. Therefore, based on the Al/Si and Ca/Si atomic ratios at each point, it is not possible to determine where the acid corrosion has taken place . However, the corroded outer area (“surface”) had a smaller average Ca/Si atomic ratio than the unaffected areas (A and B). Based on the Ca/Si atomic ratio, we could evaluate the average degree of acid attack on the whole area. The outer area (“surface”)’s average Al/Si atomic ratio was slightly smaller than those of the inner areas A and B, and the outer area had a larger Al atomic percentage than the inner areas A, B (see Figure 20). Thus, only according to the average Al/Si atomic ratio of the outer area, we cannot determine if the outer area was attacked by acid, considering the original difference in the Al/Si atomic ratio among areas.

**Figure 22:**
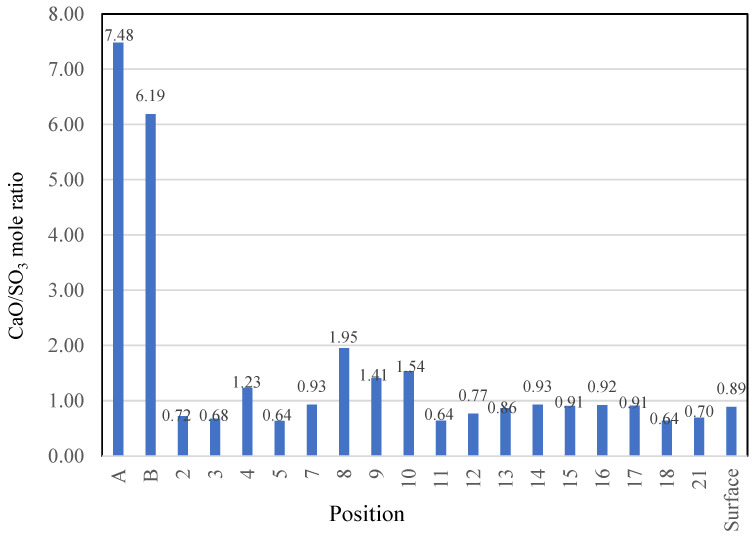
CaO/SO_3_ mole ratio of the outer and inner areas (A, B: average values of each inner area, see Figure 15b and Figure 18; Numbers: analysis results of the points in the outer area; and Surface: average value of the outer area, see Figure 19).

According to the Ca and S atomic percentages, the molar percentages of CaO and SO_3_ were calculated for the inner areas A and B, the outer area (“surface”), and the 17 points on the outer area. Their ratios are shown in Figure 22. The CaO/SO_3_ molar ratios of the two inner areas were significantly larger than those of the “surface” and the 17 points because little sulfuric acid penetrated the interior. The S element map shown in Figure 19 indicates that at points 7 and 13–17, gypsum was obviously formed, and the sulfuric acid solution undoubtedly attacked the outer area. Hence, it can be concluded that decalcification and gypsum formation occur at positions with a CaO/SO_3_ molar ratio of 0.85–1.0. The reason that gypsum was not formed in the points outside this molar ratio range was either because of less sulfuric acid penetration (the points with larger than 1.0 of CaO/SO_3_) or less calcium content (the points with smaller than 0.85 of CaO/SO_3_).

## 4. Conclusions

In this study, we investigated in detail the effects of raw materials used and mix proportions on the resistance of alkali-activated FA/GGBFS binders (AAFS) to sulfuric acid from the viewpoints of chemical and physical degradation, by measuring the dimensional and mass changes and the neutralization depth. The acid resistance of alkali-activated GGBFS/CSP (crushed stone powder) binder was also examined. The chemical compositions and the microstructures of these Ca-rich AAMs were detected by XRD and SEM-EDS after the sulfuric acid attack. The main conclusions are summarized as follows.

(1) During immersion in sulfuric acid, the Ca-rich alkali-activated binder without aggregate showed a decrease and then an increase in dimension, although the mass kept increasing. There was cracking and breakdown of the specimens, but no spalling, perhaps due to the absence of aggregate constraint.
(2) The physical degradation (scaling, spalling, cracking, breaking, etc.) and the chemical degradation (alkalinity decrease) of calcium-rich AAMs are not affected by the used raw materials and mix proportions in exactly the same way, so the physical and chemical degradation need to be considered separately when discussing the influencing factors of acid resistance of AAMs.
(3) For AAFS, heat curing and the use of a blend type of alkali activator solution (AS) of sodium silicate (SS) and NaOH can reduce both physical and chemical degradation, compared with ambient curing and the use of a sodium silicate (SS) or NaOH solution. The higher the blending ratio of SS in a blend type AS of SS and NaOH, the more likely the AAFS was to undergo physical degradation and chemical degradation. The AAFS has a higher resistance to chemical degradation when the blended AS has a higher NaOH content.
(4) The larger the liquid-filler ratio, the more severe the physical and chemical degradation of AAM. The higher the blending ratio of GGBFS in the fillers, the more severe the physical degradation of AAFS exposed to sulfuric acid, but the lesser the chemical degradation. Replacing FA in AAFS with CSP or adding BA to AAFS will increase AAM’s physical and chemical degradation.
(5) The use of retarder of sodium tartrate increases the physical and chemical degradation, but the AE only increases the chemical degradation. Using a drying shrinkage reducer composed of polyether derivatives has almost no effect on the sulfuric acid resistance of AAFS.
(6) After sulfuric acid immersion, XRD analysis can identify whether gypsum is formed in Ca-rich AAMs. The more severe the physical degradation of the Ca-rich AAMs, the higher the gypsum peak in the XRD pattern of the corroded area.
(7) For Ca-rich AAMs exposed to sulfuric acid, the dealkalization, decalcification, dealumination of C-A-S-H gels can be found from the distribution patterns of Ca, S, Al, and Na elements obtained by the SEM-EDS analysis. The average Ca/Si atomic ratio and the average Na atomic percentage in the acid corrosion area are smaller than in the unaffected area. The CaO/SO_3_ molar ratio at the location of gypsum generation ranges from 0.8 to 1.0. However, the Al atomic percentage and Al/Si atomic ratio do not demonstrate if the dealumination of the C-A-S-H gel occurs in the acid corrosion area.

As future works, we will quantitatively investigate the factors affecting physical degradation by measuring the expansion or shrinkage stress of Ca-rich AAMs exposed to sulfuric acid to determine how to produce AAMs that are less prone to spalling and cracking.

## Figures and Tables

**Table 1:** Chemical compositions and physical properties of the precursors used.

Precursors	Chemical Compositions (%, by Mass)											Specific Gravity	Blaine Value (cm^2^/g)
	SiO_2_	CaO	Al_2_O_3_	Fe_x_O_y_	Na_2_O	K_2_O	MgO	P_2_O_5_	TiO_2_	Cl	Others		
GGBFS	33.9	42.7	15.3	0.3	0.3	0.3	5.8	0.0	0.6	-	0.8	2.90	4080
FA	58.6	3.6	24.6	6.1	1.1	1.6	1.2	0.9	1.2	-	0.6	2.28	4392
CSP	58.8	9.2	14.7	7.8	3.1	2.4	2.7	0.2	0.7	-	0.4	2.73	2500
BA	40.1	24.0	16.0	4.8	4.2	2.2	1.9	2.8	1.6	0.7	1.2	2.12	3.58 *

[Notes] -: Undetected, * Fineness modulus.

**Table 2:** Characteristics of alkali activator solutions (AS).

Designation of AS	Components (Volume Ratio)	Specific Gravity	Solid Concentration by Mass (%)	Na_2_O Concentration by Mass (%)	Mole Ratio	
					SiO_2_/Na_2_O	Na/H_2_O
AS10	Diluted Na_2_SiO_3_ solution	1.352	63.0	11.4	2.10	0.101
AS01	10 mol/L NaOH solution	1.334	30.0	23.2	0.00	0.193
AS41	4 AS10 + 1 AS01	1.348	56.5	13.7	1.40	0.120
AS31	3 AS10 + 1 AS01	1.347	54.8	14.3	1.26	0.125
AS21	2 AS10 + 1 AS01	1.346	52.1	15.3	1.05	0.132

**Table 3:** Mix proportions of AAFS materials (by mass).

Series	AS	F	GGBFS:FA	AS/F	Admixture	Curing Method, Age
AS31-43	AS31	GGBFS + FA	4:6	0.43	None	Heat, 28 days
AS31-43(A)						Ambient, 28 days
AS10-43	AS10					Heat, 28 days
AS10-43(A)				0.43		Ambient, 28 days
AS/F43	AS31	GGBFS + FA	4:6	0.43	None	Heat, 7days
AS/F48				0.48		
AS/F50				0.50		
AS10-45	AS10	GGBFS + FA	4:6	0.45	None	Heat, 7days
AS41-45	AS41					
AS21-45	AS21					
AS01-45	AS01					
BFSFA	AS31	GGBFS + FA	5:5	0.40	None	Heat, 7days
BFS		GGBFS	10:0			
BFSCSP		GGBFS + CSP	4:6			
BFSFA(BA)		GGBFS + FA	4:6	0.60	BA = F × 150%	
R	AS31	GGBFS + FA	4:6	0.40	R = F × 5%	Heat, 7days
SRA					SRA = F × 3%	
AE			4:6		AE = F × 0.3%	

[Notes] AS: Alkali activator solution, F: Fillers (GGBFS, FA, CSP), AA/F: Ratio of AS to fillers, R: retarder, SRA: dry shrinkage reducing agent, AE: air-entraining agent, BA: incineration bottom ash.

## Data Availability

Data are available from the corresponding author upon reasonable request.
